# Effect of abdominal visceral fat on mortality risk in patients with severe acute pancreatitis

**DOI:** 10.1002/jgh3.12681

**Published:** 2021-11-19

**Authors:** Yu Higaki, Tsutomu Nishida, Kengo Matsumoto, Sho Yamaoka, Naoto Osugi, Aya Sugimoto, Kaori Mukai, Dai Nakamatsu, Shiro Hayashi, Masashi Yamamoto, Sachiko Nakajima, Koji Fukui, Masami Inada

**Affiliations:** ^1^ Department of Gastroenterology Toyonaka Municipal Hospital Toyonaka Osaka Japan; ^2^ Department of Gastroenterology and Internal Medicine Hayashi Clinic Suita Osaka Japan

**Keywords:** mortality, severe acute pancreatitis, visceral obesity

## Abstract

**Background and Aim:**

Obesity is a well‐known risk factor for the development and severity of acute pancreatitis (AP), but the relationship between the abdominal visceral fat area (VFA) and mortality is unclear. We evaluated the effect of the VFA on mortality in severe AP (SAP).

**Methods:**

This retrospective, single‐center cohort study examined 119 consecutive patients with SAP from April 2009 to March 2019. The VFA at the umbilical level was assessed using computed tomography. The primary endpoint was to evaluate whether visceral obesity affects mortality in SAP.

**Results:**

The median age was 63 years, and 66% of participants were male. Nine patients (7.5%) died during their hospital stay. The median body mass index (BMI) was 22.2 kg/m^2^, and six obese patients had a BMI of over 30 kg/m^2^ (5%). The median waist circumference and VFA were 85.5 cm and 112 cm^2^, respectively. Sixty‐eight (57.1%) patients had a VFA over 100 cm^2^. The prognostic factor score based on the Japanese guidelines for AP management (cutoff value [COV], 4; area under the curve [AUC] = 0.869) and age [COV, 72; AUC = 0.780]) showed moderate accuracy for predicting mortality, followed by the VFA (COV, 167 cm^2^; AUC = 0.679). Univariate logistic analysis, but not multivariate analysis, showed that an increased VFA was associated with a significantly higher odds ratio (OR) for predicting mortality (OR: 4.38, *P* = 0.0406). The survival times of SAP patients with and without an increased VFA of 167 cm^2^ were not significantly different.

**Conclusions:**

Visceral obesity did not have a significant impact on predicting mortality in patients with SAP.

## Graphical abstract

Obesity is a risk factor for acute pancreatitis (AP) development and severity, but the relationship between abdominal visceral fat and mortality is unclear. This study showed that visceral obesity did not significantly predict mortality in Japanese patients with severe acute pancreatitis.

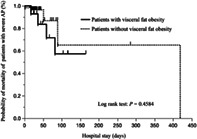

## Introduction

The incidence of acute pancreatitis (AP) has been increasing in recent years.^1^ Although a large epidemiologic study from the United States showed that mortality rates of AP declined from 12% to 2% between 1988 and 2003,^1^ patients with severe AP (SAP) still have a high mortality rate. Approximately 15–25% of patients with AP progress to moderately SAP,^2^ and a nationwide survey in Japan in 2011 showed a mortality rate of 9.5% for SAP patients.^3^

Obesity is associated with adverse alterations in adipose tissue, causing chronic inflammation, which can trigger other diseases.^4^ Adipose tissue has been identified as a potent producer of proinflammatory cytokines.^5^ Excessive cytokines derived from adipose tissue can promote the development of systemic inflammatory response syndrome (SIRS), which is thought to cause organ failure and lead to death in SAP patients. In addition, obesity is a well‐known risk factor for AP, and patients with a high body mass index (BMI) have increased morbidity and mortality.^6^, ^7^ BMI is used as a screening tool for obesity but may not always be a valid parameter for diagnosing obesity, which is characterized by the accumulation of abnormal or excessive adipose tissue, because BMI is calculated by whole body weight.

A recent systematic review of AP patients revealed a significant association between visceral fat tissue (VFT) and development/severity but did not show a relationship between VFT and mortality.^8^ In the present study, we investigated whether VFT is an additional factor in predicting mortality risk in AP patients diagnosed with SAP.

## Materials and methods

### Subjects and study design

This study was a retrospective, single‐center cohort study that examined consecutive patients with SAP at Toyonaka Municipal Hospital from April 2009 to March 2019. AP was diagnosed based on the presence of any two of the following three criteria: acute pain in the upper abdomen, elevation in serum amylase or lipase, and characteristic findings of AP upon imaging according to the Japanese guideline for the management of AP.^9^ When a diagnosis of AP was made, we repeated severity assessments within 24 h and after 24–48 h based on the severity scoring system of the Japanese Ministry of Health, Labour and Welfare according to the Japanese guideline.^9^ Briefly, the Japanese severity criteria for AP consist of two factors: (i) prognostic factors with a total point score based upon nine physiological and laboratory measurements, including bass excess, PaO_2_, blood urea nitrogen (BUN), lactate dehydrogenase (LDH), platelet count, serum calcium, C‐reactive protein (CRP), three or more positive findings in SIRS criteria, and age; and (ii) computed tomography (CT) grade determined by contrast‐enhanced CT (CE‐CT) if renal function is preserved.^9^ SAP was diagnosed when the total prognostic factor score was ≥ 3, or the CT grade was ≧2, or a combination of these was present.^9^ We enrolled patients with SAP in the present study, but we excluded patients who were diagnosed at another hospital and then transferred, or those who did not undergo CE‐CT evaluation upon admission.

### Treatment of SAP

Once we diagnosed a patient with SAP, we started supportive care at the intensive care unit (ICU) based on the Japanese guidelines.^9^ Briefly, sufficient fluid replacement and monitoring were performed within 48 h of onset, and we monitored diastolic blood pressure maintained at 65 mmHg or more and urinary output at 0.5 ml/kg/h or more. We provided pain control with fentanyl, prophylactic wide‐spectrum antibiotics within 72 h of onset, and a protease inhibitor. Local complications included acute necrotic collection (ANC), acute peripancreatic fluid collection (APFC), pancreatic pseudocyst (PPC), and walled‐off necrosis (WON) with or without infection. We performed conservative treatments for local complications. However, we applied interventional treatment to patients with infected necrotizing pancreatitis with suspected or confirmed infection accompanying an aggravated general condition based on the step‐up approach from minimally invasive procedures.

We introduced continuous regional arterial infusion (CRAI) with an admixture of biapenem and nafamostat mesylate beginning in 2010. However, the 2015 Japanese guideline stated that the efficacy of CRAI had not been confirmed, although it effectively reduced pancreatic infection and SAP mortality rates. Therefore, we stopped using CRAI for patients with SAP in 2015.

### Fat area and psoas muscle area assessment

We evaluated the pancreas using CE‐CT (Revolution GSI and Revolution EVO, GE Healthcare Japan, Tokyo, Japan) upon admission to determine the severity of AP. Using CT images, we measured the following body parameters: visceral fat area (VFA); subcutaneous fat area (SFA) at the umbilical level; and psoas muscle area at the level of the third lumbar vertebra (L3) using axial CT slices. We assessed them using the image analysis system SYNAPSE VINCENT (Fujifilm, Tokyo, Japan), which showed that VFA is colored red and SFA is colored blue, and those areas were automatically calculated (Supplementary Fig. S1). The fat area was set to a Hounsfield unit threshold of −150 to −30. We measured psoas muscle area by manual tracing. All images were measured by Y.H. The psoas muscle index (PMI) was calculated by dividing the psoas muscle area by the square of the height.^10^

### Outcomes

The primary endpoint was to evaluate the impact of VFA on predicting the in‐hospital mortality of SAP and compare the survival times of SAP patients with and without visceral obesity. Secondary endpoints were to evaluate the impact of VFA on hospital stay, ICU stay, and local complications.

### Statistical analysis

The medians and interquartile ranges (IQRs) are reported for continuous variables. Categorical variables are summarized as frequencies (percentages). We used the values of the potential risk factors measured at admission to predict mortality. We performed receiver operating characteristic (ROC) curve analysis to determine the cutoff values (COVs) for predicting mortality in SAP patients. Then, we used a univariate logistic regression model to obtain odds ratios (ORs) with 95% confidence intervals (CIs) between two groups divided by the COVs for the factors significant for predicting mortality. Using the factors that were significant in the univariate analysis, we performed multivariate logistic analysis. The cumulative survival curves of SAP patients with and without visceral obesity were compared using the log‐rank test.

All reported *P*‐values were two‐sided, and *P* < 0.05 was considered significant. Statistical analyses were performed with JMP statistical software (ver. 15.2.1, SAS Institute, Inc., Cary, NC, USA).

## Results

### Baseline characteristics and clinical course

A total of 444 patients were diagnosed with AP and hospitalized in our hospital during the study period. Of them, 120 patients (27%) were diagnosed with a severe case, but one patient did not undergo CE‐CT upon admission. Ultimately, 119 patients were enrolled in this study. The baseline characteristics of the study patients are shown in Table 1. The median age was 62 years (IQR 50, 74), and 66% of the patients were male. The cause of AP was alcohol in 50 patients (42%), cholelithiasis in 37 patients (31%), idiopathic disease in 11 patients (9%), and others. The median BMI was 22.2 kg/m^2^, and six obese patients had a BMI over 30 kg/m^2^ (5%). The median waist circumference, VFA, and SFA were 85.5 cm, 112 cm^2^, and 114 cm^2^, respectively. There were 68 (57%) patients with a VFA over 100 cm^2^, indicative of the risk of obesity‐related disorders in Japanese people as a diagnostic criteria of obesity.^11^ The psoas muscle area at the third lumbar vertebra (L3) level was 12.5 cm^2^, and the PMI was 4.7 kg/m^2^.

**Table 1:** Characteristics of patients with severe acute pancreatitis

	*N* = 119
Age (IQR), years	62 (50–74)
Male sex, *n* (%)	79 (66.4)
Etiology	
Alcohol, *n* (%)	50 (42.0)
Cholelithiasis, *n* (%)	37 (31.1)
Pancreatolith, *n* (%)	6 (5.0)
Post‐ERCP, *n* (%)	4 (3.4)
Drugs, *n* (%)	3 (2.5)
Hyperlipidemia, *n* (%)	3 (2.5)
Idiopathic disease, *n* (%)	11 (9.2)
Others, *n* (%)	5 (4.2)
Body parameters	
Body weight (IQR), kg	60 (52–70)
Height (IQR), cm	165 (154.2–171)
BMI (IQR), kg/m^2^	22.2 (20.6–24.6)
BMI ≥ 30 kg/m^2^, *n* (%)	6 (5.0)
Waist circumference (IQR), cm	85.5 (79.8–91.8)
VFA at the umbilical level (IQR), cm^2^	112 (65.7–165)
VFA at the umbilical level ≥ 100 cm^2^, *n* (%)	68 (57.1)
SFA at the umbilical level (IQR), cm^2^	114 (81.2–159)
VFA at L3 level (IQR), cm^2^	110 (57, 180)
VFA at L3 level ≥ 100 cm^2^, *n* (%)	65 (54.6)
SFA at L3 level (IQR), cm^2^	92 (64, 130)
Psoas muscle area, cm^2^	12.5 (9.54–16.3)
Psoas muscle index (IQR), cm^2^/m^2^	4.7 (3.8–5.5)

BMI, body mass index; ERCP, endoscopic retrograde cholangiopancreatography; IQR, interquartile range; PMA, psoas muscle area; SFA, subcutaneous fat area; VFA, visceral fat area.

The severity outcomes of the study patients are shown in Table 2. According to the Japanese guidelines, the median prognostic factor score and CT severity score were 1 and 2, respectively. The median ICU stay and hospital stay were 4 and 29 days, respectively. During the hospital stay, 33 patients (28%) developed local complications, including 2 APFC, one ANC, 14 PPC, and 16 WON. Nine patients (7.5%) died during their hospital stay.

**Table 2:** Severity and clinical outcomes of patients with severe acute pancreatitis

Outcome	*N* = 119
Severity outcomes	
Prognostic factor score, median (IQR)	1 (0.25. 3)
0, *n* (%)	31 (26.1)
1, *n* (%)	37 (31.1)
2, *n* (%)	19 (16.0)
3, *n* (%)	16 (13.4)
4 or more, *n* (%)	16 (13.4)
CE‐CT grade	
Grade 1, *n* (%)	10 (8.4)
Grade 2, *n* (%)	98 (82.4)
Grade 3, *n* (%)	11 (9.2)
Clinical outcomes, median (IQR)	2 (2, 2)
ICU stay (IQR) days	4 (2, 8)
ICU stay ≥ 10 days, *n* (%)	22 (18.5)
Hospital stay (IQR) days	29 (20, 44)
Hospital stay ≥ 30 days, *n* (%)	43 (36.1)
Hospital mortality, *n* (%)	9 (7.6)
Treatment	
Enteral nutrition, *n* (%)	79 (66.3)
Continuous regional arterial infusion, *n* (%)	23 (19.3)
EUS‐CD, *n* (%)	5 (4.6)
Percutaneous drainage, *n* (%)	5 (4.6)
Continuous hemodiafiltration, *n* (%)	2 (1.8)
Plasmapheresis, *n* (%)	1 (0.9)
Complication	
Local complication, *n* (%)	33 (27.7)
Infected/sterile, *n* (%)	13 (10.9)/20 (18.5)
Acute peripancreatic fluid collection, *n* (%)	2 (1.7)
Acute necrotic collection, *n* (%)	1 (0.84)
Pancreatic pseudocyst, *n* (%)	14 (11.8)
Walled‐off necrosis, *n* (%)	16 (13.4)
Thrombogenic events, *n* (%)	10 (18.2)

CE‐CT, contrast‐enhanced computed tomography; EUS‐CD, endoscopic ultrasonography‐guided pancreatic cyst drainage; ICU, intensive care unit; IQR, interquartile range.

### Risk factors predicting mortality in SAP patients

We evaluated the COVs of these clinical and body parameters for predicting mortality via ROC curve analysis. We calculated the COVs of the prognostic factor score, age, BMI, waist circumference, VFA, SFA, psoas muscle area, and PMI, which were 4, 72 years, 20.5 kg/m^2^, 88.8 cm, 167 cm^2^, 143 cm^2^, 7.2 cm^2^, and 5.32 kg/m^2^, respectively. The prognostic factor score and age showed moderate accuracy over 0.7 of the area under the curve, followed by VFA (AUC = 0.679), but BMI, waist circumference, SFA, psoas muscle area, and PMI had low accuracies in predicting mortality for SAP patients (Table 3).

**Table 3:** Cutoff values, sensitivity, and specificity of clinical factors predicting mortality for patients with severe acute pancreatitis

	COV	AUC	Sensitivity	Specificity
Prognostic factor score	4	0.869	0.667	0.909
Age, years	72	0.780	0.889	0.736
BMI, kg/m^2^	20.5	0.537	1.00	0.246
Waist circumference, cm	88.8	0.538	0.778	0.640
VFA at the umbilical level, cm^2^	167	0.679	0.556	0.736
SFA at the umbilical level, cm^2^	143	0.521	1.00	0.346
VFA at the L3 level, cm^2^	158	0.669	0.667	0.709
SFA at the L3 level, cm^2^	105	0.561	0.889	0.373
Psoas muscle area, cm^2^	7.2	0.564	1.00	0.291
Psoas muscle index	5.32	0.548	1.00	0.300

AUC, area under the curve; BMI, body mass index; COV, cutoff value; PMA, psoas muscle area; SFA, subcutaneous fat area; VFA, visceral fat area.

**Figure 1:**
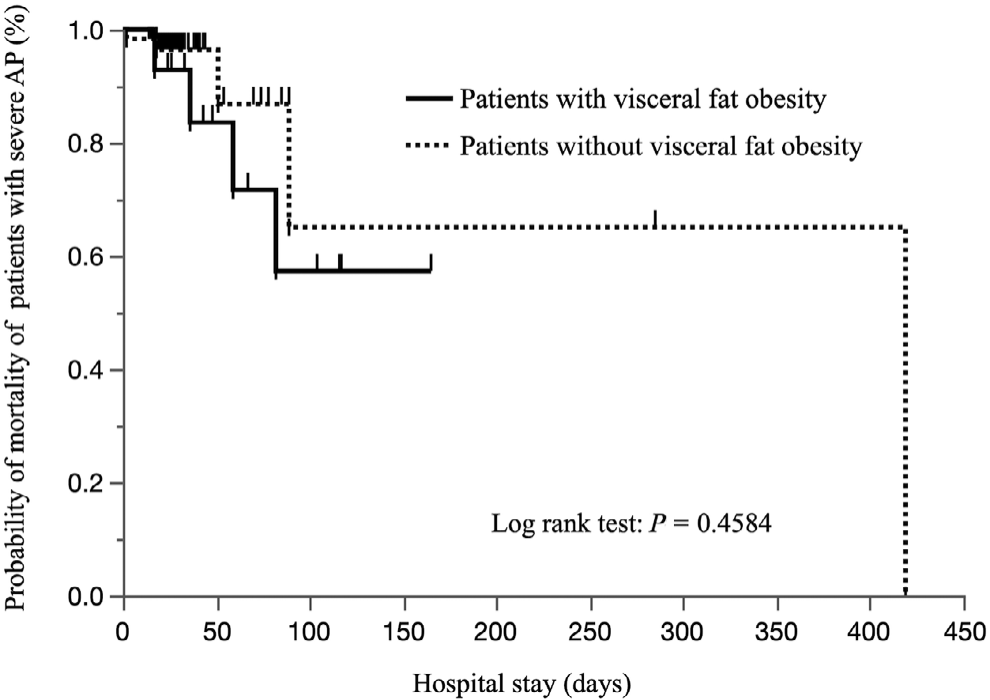
Overall survival curves in severe acute pancreatitis (AP) patients with and without a higher VFA, which were not significantly different.

Univariate logistic analysis using clinical factors, including the prognostic factor score, age, and VFA divided by COVs, showed that higher prognostic factor scores (OR: 20.0, 95% CI, 4.33–92.4, *P* = 0.00001), older age (OR: 22.3, 95% CI, 2.68–186, *P* = 0.0041), and larger VFA (OR: 4.38, 95% CI, 1.67–18.0, *P* = 0.0406) were significant in predicting mortality. Then, we performed multivariate analysis using three significant factors. The prognostic factor score and age, but not VFA (*P* = 0.4258), were significant independent factors (Table 4). A plot of the survival rates of the 119 SAP patients with and without visceral obesity and a VFA of over 167 cm^2^ is shown in Figure 1. There was no significant difference between them.

**Table 4:** Risk factors associated with mortality in patients with severe acute pancreatitis: univariate and multivariate logistic regression analysis

	Univariate		Multivariate	
	Odds ratio (95% CI)	*P*‐value	Odds ratio (95% CI)	*P*‐value
Prognostic factor score ≥ 4	20.0 (4.33–92.4)	0.00001	8.57 (1.57–46.6)	0.0130
Age ≥ 72 years	22.3 (2.68–186)	0.0041	12.5 (1.37–114)	0.0251
VFA at the umbilical level ≥ 167 cm^2^	4.38 (1.67–18.0)	0.0406	2.04 (0.353–11.8)	0.4258

VFA, visceral fat area.

Similarly, we evaluated whether those significant risk factors were associated with an ICU stay ≥ 10 days and a hospital stay ≥ 30 days in patients with SAP. The univariate logistic analysis showed that the prognostic factor score and larger VFA were significant for ICU stays ≥ 10 days and older age was significant for hospital stays ≥ 30 days. Multivariate logistic regression analysis revealed that the prognostic factor score was a significant risk factor for ICU stays ≥ 10 days and VFA was borderline significant (*P* = 0.0707) (Table 5).

**Table 5:** Risk factors associated with a hospital stay ≥ 30 days in patients with severe acute pancreatitis, univariate and multivariate logistic regression analysis

	Univariate		Multivariate	
	Odds ratio (95% CI)	*P*‐value	Odds ratio (95% CI)	*P*‐value
ICU stay ≥ 10 days				
Prognostic factor score ≥ 4	4.56 (1.47–14.1)	0.0084	3.46 (1.05–11.4)	0.0418
Age ≥ 72 years	1.34 (0.507–3.54)	0.5549		
VFA ≥ 167 cm^2^	3.69 (1.30–10.5)	0.0145	2.79 (0.917–8.46)	0.0707
Hospital stay ≥ 30 days				
Prognostic factor score ≥ 4	2.61 (0.895–7.60)	0.0789		
Age ≥ 72 years	3.02 (1.35–6.76)	0.0073		
VFA ≥ 167 cm^2^	2.27 (0.873–5.89)	0.0926		

ICU, intensive care unit; VFA, visceral fat area.

### Local complications and clinical and body parameters

Of the 33 patients affected by local complications, 13 had infections and 20 did not. Moreover, older patients with complications tended to have infections (*P* = 0.0645), but there was no relationship between the prognostic factor score, age, and VFA and local complications (Table 6).

**Table 6:** Details of local complications based on the Japanese severity score (JSS), age, and VFA

Local complication	Total		With			Without	*P*‐value^‡^
		Total	Infected	Sterile	*P*‐value^†^		
Prognostic factor score							0.0019
≥ 4	16	10	5	5	0.4611	6	
< 3	103	23	8	15		80	
Age (years)							0.8257
≥ 72	37	11	7	4	0.0645	26	
< 72	82	22	6	16		60	
VFA (cm^2^)							1.000
≥ 167	21	6	3	3	0.6588	15	
< 167	98	27	10	17		71	

^†^ Compared with infected and sterile local complications.

^‡^ Compared with with and without local complications.

## Discussion

We found that visceral obesity did not have a significant additional impact on the mortality of 119 Japanese patients with SAP compared with the prognostic factor score and age, and there was no significant difference in survival, although visceral obesity may increase the length of ICU stay. A recent systematic review revealed that 9 of 11 studies showed a significant association between VFA and severity. On the other hand, only 2 of 11 studies showed a significant association between VFA and mortality, but the others did not show any association or the data were not available.^8^ Therefore, whether VFA can affect mortality in AP patients remains unclear, but it did not seem to have a strong impact.

Several studies have shown that obesity or intra‐abdominal fat observed with CT, overweight, and BMI are independent risk factors for SAP, severity, local complications, or death^6^, ^7^, ^12^, ^13^, ^14^; moreover, some guidelines suggest obesity as an important prognostic factor,^15^ and BMI over 30 kg/m^2^ increases mortality and progression to severe disease.^9^, ^16^ In Asian countries, very few patients have BMI values that are too high; there seemed to be no association between high BMI and SAP, and even adding a factor of BMI did not improve the prediction of the severity of AP.^17^ According to a nationwide epidemiological study in Japan, the number of patients with a BMI over 30 kg/m^2^ was 25 out of 825 patients with AP (3%), and among these obese patients, only one death occurred.^18^ In the present study, we had only six patients (5%) with a BMI over 30 kg/m^2^, and these patients did not die. In addition, we compared 25 patients with a BMI over 25 kg/m^2^ and 94 with a lower BMI, which revealed no difference in mortality (4.0 *vs* 8.5%, *P* = 0.6825) because the Japan Society for the Study of Obesity defines a BMI over 25 kg/m^2^ as obese for the Japanese population based on numerous epidemiological studies. Furthermore, the BMI COV (20.5) had low accuracy in predicting mortality in SAP patients. We concluded that there was no additional impact of increased BMI on mortality risk in Japanese SAP patients compared with the prognostic factor score.

Adipose tissue itself has been shown to cause inflammatory changes,^19^ which may exacerbate inflammation due to AP. The release of inflammatory cytokines from adipocytes is expected to cause SIRS and increase local complications, although the mechanism is not clear. Recently, de Oliveira *et al*. reported, in a mouse study, that pancreatic lipase injected into VFT in the absence of adipocyte triglyceride lipase hydrolyzed adipose triglycerides and generated excess nonesterified fatty acids, which caused organ failure in the absence of AP.^20^ Therefore, we focused on VFT for the mortality risk of SAP patients with high mortality instead of BMI. Previously, the Japanese Visceral Fat Syndrome Study Committee of the Ministry of Health and Welfare of Japan established a VFA COV of 100 cm^2^. In the present study, we had 68 (57%) patients with visceral obesity with a VFA over 100 cm^2^, which was not a higher prevalence than the 62% of 5347 asymptomatic Japanese individuals with a mean age of 54 years.^21^ The mortality rate of obese patients was similar to that of patients with VFAs less than 100 cm^2^ (8.8% *vs* 5.9%, *P* = 0.7307). Although we used the higher VFA value of 167 cm^2^ to predict mortality, which showed a significantly higher OR of 4.38 (*P* = 0.0406) for predicting mortality by univariate logistic analysis, there was no difference when using multivariate analysis and the log‐rank test. However, when using a higher VFA as the COV, a higher VFA had a borderline significant mortality risk relative to that of a lower VFA (19.1% *vs* 5.1%, *P* = 0.0505), which may be caused by the small number of patients.

There are several limitations due to the nature of this single‐center, retrospective study. First, the number of SAP patients was small for the multivariate analysis or ROC curve, which may cause overfitting and limited generalizability of the study results. In the present study, the VFA measured by CT did not have a significant additional impact on mortality, but SAP patients with a VFA of 167 cm^2^ had higher mortality than those with a VFA of less than 167 cm^2^. An analysis including additional SAP patients may show the impact of VFA on mortality. Therefore, we might underestimate the VFA, but it does not seem to have a higher impact than the prognostic factor score or age. Second, we determined the severity of AP according to the Japanese severity criteria for AP—not the Acute Physiology and Chronic Health Evaluation II (APACHE II) score^22^ or the modified Marshall scoring system, which is endorsed in the revised Atlanta classification^23^—because we use the Japanese guidelines in regular clinical practice. However, a recent multicenter validation study with 1159 Japanese AP patients revealed that the prognostic factor score has the ability for predicting mortality equivalent to that of the APACHE II score.^24^ This validation study also showed that the AUC of the prognostic factor score for predicting severity according to the revised Atlanta classification was 0.83 (95% CI, 0.81–0.86), which was significantly higher than the AUC (0.80, 95% CI, 0.77–0.82) of APACHE II (*P* = 0.001). Therefore, we considered that the prognostic factor score of the Japanese guidelines was feasible for evaluating the impact of visceral fat in predicting mortality in Japanese AP patients. Third, we used the VFA data at a single level, namely the umbilical level, but not the total volume or VFA data at the L3 level. Applying the total volume is not clinically feasible because of its time‐consuming nature, and the umbilical level is the same as the waist circumference, which offers the maximum ratio of adipose tissue to total tissue area.^25^ We also evaluated the COV of VFA at the L3 level, which was 158 cm^2^, and its AUC was similar to that at the umbilical level (AUC: 0.669) (Table 3). Therefore, we ultimately used fat measurements at the umbilical level.

In conclusion, we found that abdominal visceral obesity, which was evaluated by the VFA at the umbilical level, did not have a significant additional impact on predicting mortality compared with the prognostic factor score and age for the Japanese population with SAP.

### Statement of ethics

The present study was conducted in accordance with the Declaration of Helsinki, and approval was obtained from the Institutional Review Board of Toyonaka Municipal Hospital on 6 January 2021 (No. 2021‐01‐01). The requirement for informed consent was waived via the *opt‐out* method on our hospital website.

## Supporting information


**Supplementary Figure S1.** Measurements of the visceral fat area (VFA) and subcutaneous fat area (SFA). Measured VFA (red) and SFA (blue) at the umbilical level. They were automatically assessed by computed tomography using the SYNAPSE VINCENT image analysis system (Fujifilm, Tokyo, Japan).

## Data Availability

The data that support the findings of this study are available on request from the corresponding author. The data are not publicly available due to restrictions (i.e. they contain information that could compromise the privacy of the research participants).
