# Supplementary figures and images for: Effect of abdominal visceral fat on mortality risk in patients with severe acute pancreatitis
Source: JGH Open. 2021 Nov 19;5(12):1357–62. doi: 10.1002/jgh3.12681 (PMC8674542; doi:10.1002/jgh3.12681)

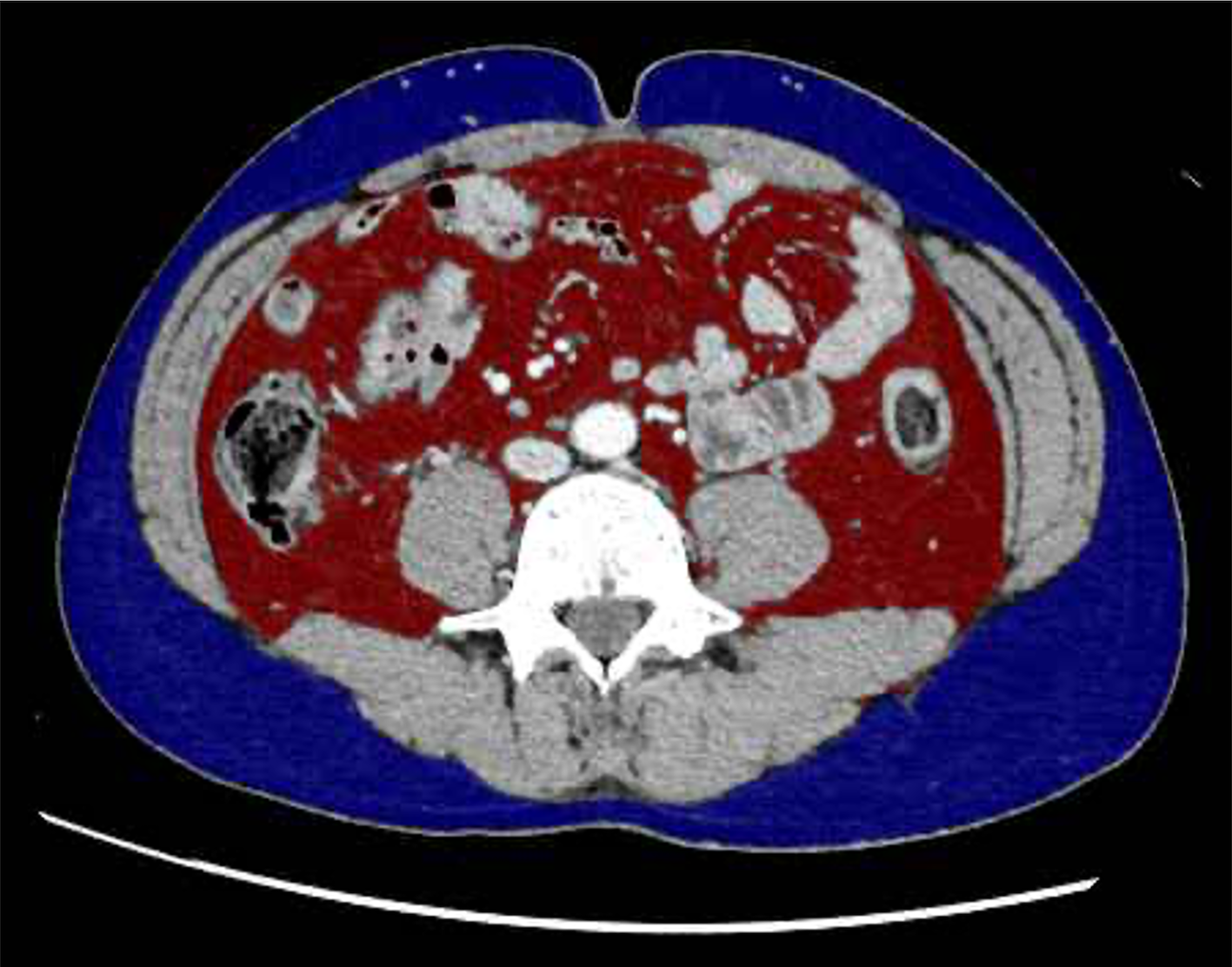

Supplement: Supplementary file 1 — Supplementary Figure S1. Measurements of the visceral fat area (VFA) and subcutaneous fat area (SFA). Measured VFA (red) and SFA (blue) at the umbilical level. They were automatically assessed by computed tomography using the SYNAPSE VINCENT image analysis system (Fujifilm, Tokyo, Japan). [file JGH3-5-1357-s001.tiff]
